# Keratoconus Diagnosis: Validation of a Novel Parameter Set Derived from IOP-Matched Scenario

**DOI:** 10.1155/2020/6530279

**Published:** 2020-10-28

**Authors:** Dan Lin, Lei Tian, Shu Zhang, Like Wang, Ying Jie, Yongjin Zhou

**Affiliations:** ^1^Shenzhen University, Health Science Center, School of Biomedical Engineering, Xueyuan Avenue 1066, Shenzhen 518055, China; ^2^Beijing Institute of Ophthalmology, Beijing Tongren Eye Center, Beijing Tongren Hospital, Capital Medical University, Beijing, China; ^3^Beijing Ophthalmology & Visual Sciences Key Laboratory, National Engineering Research Center for Ophthalmology, Beijing 100730, China; ^4^Beijing Advanced Innovation Center for Big Data-Based Precision Medicine, Beihang University and Capital Medical University, Beijing Tongren Hospital, Beijing 100730, China

## Abstract

**Purpose:**

Considering that intraocular pressure (IOP) is an important confounding factor in corneal biomechanical evaluation, the notion of matching IOP should be introduced to eliminate any potential bias. This study aimed to assess the capability of a novel parameter set (NPS) derived from IOP-matched scenario to diagnose keratoconus.

**Methods:**

Seventy samples (training set; 35 keratoconus and 35 normal corneas; pairwise matching for IOP) were used to determine NPS by forward logistic regression. A large validation dataset comprising 62 matching samples (31 keratoconus and 31 normal corneas) and 203 unmatching samples (112 keratoconus and 91 normal corneas) was used to evaluate its clinical significance. To further assess its diagnosis capability, NPS was compared with the other two prior biomechanical indexes.

**Results:**

NPS was comprised of three biomechanical parameters, namely, DA Ratio Max 1 mm (DRM1), the first applanation time (AT1), and an energy loading parameter (Eload). NPS was successfully applied to the validation dataset, with a higher accuracy of 96.8% and 95.6% in the IOP-matched and -unmatched scenarios, respectively. More surprisingly, accuracy of NPS was 95.5% in the combined validation, an improvement compared to the two prior biomechanical indexes.

**Conclusions:**

This is the first study taking IOP bias into consideration to determine a biomechanical parameter set. Our study shows that NPS indeed offers comparable performance in keratoconus diagnosis. *Translational Relevance*. Determining a parameter set after eliminating the influence from IOP is useful in revealing the essential differences between keratoconus and normal corneas and possibly facilitating further progress in keratoconus diagnosis.

## 1. Introduction

The clinical diagnosis of keratoconus remains a significant challenge, especially before the appearance of any signs or symptoms. It has been well documented that the changes in the biomechanical properties of keratoconus are postulated to occur before the disease becomes tomographically apparent [1–4]. These changes can be certainly attributed to the abnormalities in stromal collagen [1–4]. Therefore, it is becoming increasingly popular to detect keratoconus with the biomechanical parameters derived from Corneal Visualization Scheimpflug Technology (Corvis ST, Oculus, Germany).

Corvis ST is a relatively new device that induces corneal deformation with an air puff and allows the real-time monitoring of the entire deformation process using an ultra-high-speed Scheimpflug camera [5, 6]. During the dynamic process of corneal deformation, Corvis ST can be used to simultaneously measure the corneal biomechanical parameters and intraocular pressure (IOP). More recently, IOP has been gradually accepted as a biasing factor for corneal biomechanical evaluation [7, 8], and thus it is supposed to be excluded to ensure an unbiased analysis. Unfortunately, in the field of keratoconus diagnosis, there is a lack of studies that determine the combined biomechanical parameters from the perspective of IOP matching. Additionally, plethora of new and important biomechanical parameters [5, 9] is catching up, but prior studies [10–12] have not considered them yet.

To make matters more precise and comprehensive, it is our job to develop a novel parameter set (NPS) taking IOP bias and new biomechanical parameters into consideration. As we will show later, adopting this new parameter set allows clinicians to diagnose keratoconus better and easier.

## 2. Patients and Methods

### 2.1. Patients

A total of 335 corneal samples were included in this study, which are divided into two groups: keratoconus group (*n* = 178) and normal cornea group (*n* = 157). Among them, 132 corneal samples (66 keratoconic corneas and 66 normal corneas) were pairwise matched for IOP, while the remaining 203 eyes (112 keratoconic corneas and 91 normal corneas) were not matched for IOP. The maximum IOP difference between the pairs was 0.6 mm Hg. 70 corneas with IOP-matched scenario were randomly selected for the assessment of parameter set. Moreover, 62 and 203 corneas with IOP-matched and IOP-unmatched scenarios, respectively, were used to assess the performance of the parameter set. For patients diagnosed with keratoconus in only one eye, the particular eye was selected for measurement. Meanwhile, one eye was randomly selected from normal controls and patients with keratoconus in both eyes.

All patients underwent a complete ophthalmic examination, including a detailed assessment of uncorrected distance visual acuity, corrected distance visual acuity, slit-lamp microscopy and fundus examination, corneal topography (Allegro Topolyzer; WaveLight Laser Technologie AG, Erlangen, Germany), corneal tomography (Pentacam; Oculus Optikgeräte GmbH), ocular biomechanics, and IOP measurement (Corvis ST). All measurements were performed by two experienced ophthalmologists in a single visit. A diagnosis of keratoconus was carried out if the eye had (i) an irregular cornea, determined by distorted keratometry mires or distortion of the retinoscopic or ophthalmoscopic red reflex, and (ii) at least one of the following slit-lamp signs: Vogt's striae, Fleischer's ring with an arc >2 mm, or corneal scarring consistent with keratoconus [13–15].

Potential subjects were excluded from this study if they (i) had previously undergone corneal or ocular surgery, (ii) had ocular pathology other than keratoconus, and/or (iii) had systemic diseases that affect their eye. All participants were asked to remove soft contact lenses for at least 2 weeks and rigid contact lenses for at least 1 month prior to the examination. Clinical data were collected in 2018 at the Beijing Institute of Ophthalmology, Beijing Tongren Hospital, Beijing, China. All participants signed a written informed consent form, in accordance with the ethical principles stated in the Declaration of Helsinki.

### 2.2. Collection of Parameters

A total of 21 biomechanical parameters were extracted, including 11 parameters from the Corvis ST software, 9 parameters proposed by our group previously, and 1 parameter defined by other scholars.

Corvis ST allows the noninvasive imaging of the cornea's dynamic deformation in response to an air puff. A high-speed Scheimpflug camera records the movements of the cornea and then displays them on the built-in control panel in a slow motion. During the deformation response, a precisely metered air pulse causes the cornea to move inward or flatten (the phenomena of corneal applanation), which is known as the first applanation (A1). The cornea continues to move inward until reaching a point with highest concavity. After that, it rebounds from this concavity to another point of applanation (A2) and then returns to its normal convex curvature. After completing the deformation process, several output measurements are generated from Corvis ST. All these parameters and their details are listed in Table 1.

Our research team has proposed several new parameters to measure the biomechanical behavior of corneas [9]. For instance, maximum area of deformation (MA) is used to describe the maximum corneal deformation area within the two knees. Maximum area–time of deformation (MA-time) represents the time from the beginning of deformation to the occurrence of maximum deformation area. Corneal contour deformation (CCD) describes a distance between the original contour and the contour with the highest concavity. Maximum corneal inward/outward velocity (*V*_inmax_/*V*_outmax_) represents the maximum corneal inward/outward deformation velocity at centerline. In 2016, we subsequently proposed energy absorbed area (*A*_absorbed_) and Tangent stiffness coefficient (*S*_TSC_) to indicate the corneal viscosity and elasticity, respectively [5]. Additionally, the corneal viscoelasticity is defined by both energy loading (Eload) and energy return (Ereturn) of cornea during the air puff indentation.

Stiffness parameter (SP-A1) is a parameter associated with corneal stiffness [16], which has been defined as resultant pressure (Pr) divided by the amplitude of deformation at A1. Pr is defined as the adjusted pressure at A1 (adj-AP1) minus a biomechanically corrected IOP (bIOP) [17]. The computational formula is as follows: SP-A1 = (adj-AP1 − bIOP)/deformation amplitude at A1.

### 2.3. Statistical Analysis

Statistical analyses were performed using R (RStudio 3.4.0). The Kolmogorov–Smirnov test was used to assess the normality of data. Both Welch's modified Student's two-sample *t*-test and Mann–Whitney *U* test were applied to compare the difference of biomechanical parameters between keratoconus and normal groups. *P* values less than 0.05 were considered statistically significant.

Forward logistic regression was performed to determine NPS based on all the biomechanical parameters in IOP-matched scenario. Area under the Curve of ROC (AUC), F1 score, sensitivity, specificity, and accuracy were calculated to evaluate the discriminative ability of parameter sets. The values closer to 1 indicate a greater performance.

## 3. Results

In the present study, bIOP was used to correct IOP based on the finite element modeling [17]. As shown in Table 2, all parameters were significantly different between keratoconus and normal cornea groups (*P* < 0.05), except for bIOP and age.

As a consequence, the three significantly differential parameters, namely, DRM1, AT1, and Eload, were selected for NPS. The equations are presented as follows:

(1) $$\begin{array}{c} \text{Beta}=\left(\left(\mathrm{A1*DRM1}\right)+\left(\text{A}2*\text{AT}1\right)+\left(\text{A}3\mathrm{*Eload}\right)+\text{A}4\right),\\ \text{Possibility}=\frac{\exp\left(\text{Beta}\right)}{\left(1+\exp\left(\text{Beta}\right)\right)}. \end{array}$$

**Figure 1:**
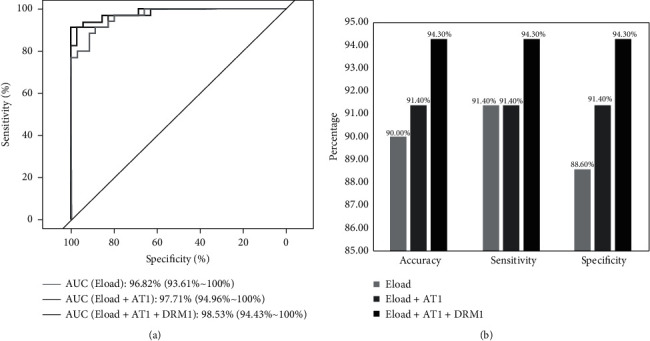
(a) Receiver operator characteristic curve for each step of the forward logistic regression in IOP-matched scenario (35 keratoconus and 35 normal eyes). (b) Gain in sensitivity and specificity with each step of the logistics regression to establish the novel NPS with IOP-matched scenario (35 keratoconus and 35 normal eyes; best cut-off point = 0.5).

The impact of each NPS parameter was evaluated, as demonstrated in Figure 1. When Eload was included in the logistic regression model, the AUC of NPS was found to be 96.8%, with a sensitivity of 91.4% and specificity of 88.6%. Following the addition of AT1, the AUC and specificity of NPS were improved to 97.7% and 91.4%, respectively. Lastly, after the inclusion of DRM1, NPS exhibited the highest AUC, accuracy, sensitivity, and specificity of 98.5%, 94.3%, 94.3%, and 94.3%, respectively, at the optimal cut-off point of 0.5.

**Figure 2:**
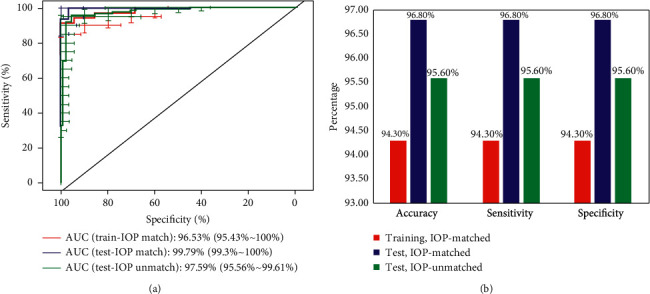
Representative graphs for ROC curves (a) and three evaluation indicators (accuracy, sensitivity, and specificity) (b) for NPS in both IOP-matched training set (35 keratoconus and 35 normal eyes), and IOP-matched (31 keratoconus and 31 normal eyes) and IOP-unmatched (112 keratoconus and 91 normal eyes) validation.

The performance of NPS in both training set and validation set was illustrated in Figure 2. Interestingly, NPS performed better in the validation set (accuracy = 96.8% and 95.6% in IOP-matched and -unmatched validation, respectively) than in the training set (accuracy = 94.3%). More notably, in both training set and validation set, NPS exhibited the same score of three evaluation indicators at the best cut-off point of 0.5.

**Figure 3:**
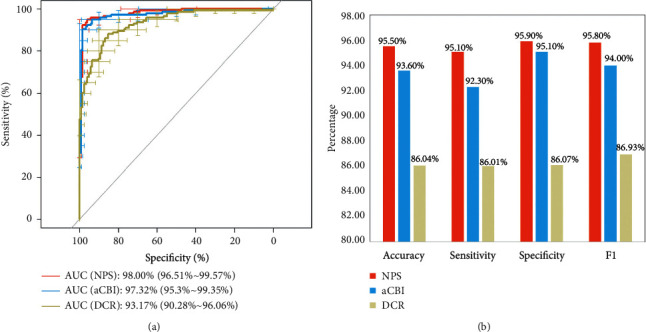
Representative graphs for ROC curves (a) and four evaluation indicators (accuracy, sensitivity, specificity, and F1) (b) of NPS, aCBI, and DCR in combined (145 keratoconus and 122 normal eyes) validation.

Likewise, in the combined validation, NPS showed a comparable diagnosis capability compared to two reported parameter sets, namely, adjusted Corvis Biomechanical Index (aCBI) [11] and Dynamic Corneal Response Index (DCR) [12]; see Figure 3. The AUC of NPS (98.0%) was slightly higher than the other two parameter sets (aCBI: 97.3%; DCR: 93.2%). At the optimal cut-off point of 0.5, the accuracy of NPS reached 95.5%, while it reached 93.6% for aCBI and 86.0% for DCR.

## 4. Discussion

With the confounding influence of IOP being discovered gradually, how are we supposed to diagnose keratoconus using biomechanical parameters from Corvis ST videos? Vinciguerra et al. [10] have reported that the parameter set determined from IOP-unmatched dataset and validated with IOP-matched scenario [11] can result in decreased accuracy. From our point of view, this decline is attributed not only to the matching of central corneal thickness (CCT) as presumed by the authors [11], but also to the fact that the parameter set is established from the IOP-unmatched dataset. Therefore, in this study, a dataset independent of IOP was deliberately used to determine the parameter set with no biasing effect of IOP. Moreover, two additional datasets of both IOP-influenced and IOP-uninfluenced were used to validate the discriminative ability of NPS. Interestingly, the results demonstrated a slightly improved accuracy as well as the balanced indicators of NPS (Figure 2) during the validation step, which in turn confirmed the strategy of this study.

It has been noted that the biomechanical parameters reported in prior studies [10–12] are limited to those available from Corvis ST software. In the present study, we incorporated more parameters from different sources [5, 9] to enable a comprehensive analysis. As presented in Figure 1, only 3 parameters (i.e., DRM1, Eload, AT1) were ultimately selected in IOP-matched group, in which Eload is obtained from an external data source [5]. This result indicated that new biomechanical parameters should be paid more attention as they may provide valuable knowledge in terms of keratoconus detection.

As the main goal of our study was to validate whether a parameter set derived from IOP-matched scenario can exhibit its clinical significance in keratoconus diagnosis, comparing it with the established biomechanical indexes can help to achieve this goal. The results showed that NPS exhibited similar or better performance in the validation set (Figure 3). In our opinions, these may be primarily caused by the following four reasons. First, NPS incorporates a new biomechanical parameter, namely, Eload [5]. As a parameter describing energy loading during corneal deformation, Eload reflects the viscoelasticity, a significant biomechanical property of corneal tissues, thereby playing a big role in the diagnosis. Second, DRM1 is a well-known biomechanical parameter representing the ability of deformation. It makes an impact on keratoconus screening, which is consistent with prior studies [10–12]. Third, in general, the simpler the model, the greater the robustness. NPS contains only 3 parameters, which is less than aCBI or DCR, making it practical to adjust to each new dataset. Finally, the biomechanical parameters of NPS are chosen on the basis of IOP-matched scenario and hence are naturally stable and can be used to reveal the true difference between keratoconus and normal corneas. Given all these reasons, findings from this study could be interpreted to some extent.

It is worth noting that we deliberately did not match CCT, another confounding factor in corneal biomechanics [7], because we believed that, as an important part in the pathogenesis of keratoconus, corneal thinning should be included to reveal the resulting changes in biomechanical stability. Nevertheless, the potential limitation of this study is the lack of gold standard for measuring IOP as well as the appropriate statistical test for evaluating indicators of these three biomechanical indexes (Figure 3). While there are signs of improvement, the results should be interpreted cautiously.

In conclusion, this study indicated that the parameter set (NPS) derived from IOP-matched scenario can effectively differentiate keratoconus from normal corneas. Using this parameter set prevents us from unnecessarily considering the confounding influence from IOP. It will lead to the ease of use in the clinical practice and the follow-up diagnosis success at earlier stages of keratoconus. Further research is warranted to further elucidate its potential use.

## Figures and Tables

**Table 1:** Biomechanical parameters derived from the Corvis ST software and their corresponding definitions.

Parameters	Abbreviation	Definitions
The time of the first applanation (ms)	AT1	The length of time from the initiation of air puff to the first applanation
The time of the second applanation (ms)	AT2	The length of time from the initiation of air puff to the second applanation
The length of the first applanation (mm)	AL1	The lengths of flattened cornea at the first applanation
The length of the second applanation (mm)	AL2	The lengths of flattened cornea at the second applanation
The velocity of the first applanation (m/s)	*V*_in_	Speed of corneal apex at the first applanation
The velocity of the second applanation (m/s)	*V*_out_	Speed of corneal apex at the second applanation
The time of highest concavity (ms)	HC-time	Time from the initiation of air puff until the highest concavity of the cornea
DA Ratio Max 1 mm	DRM1	Maximum ratios of corneal deformation at the apex divided by the average deformation 1 mm to either side of the apex
Peak distance (mm)	PD	Distance of the two knees at highest concavity
Deformation amplitude (mm)	DA	Maximum deformation amplitude at highest concavity
The highest radius of concavity (mm)	HC-radius	Corneal concave curvature at its highest concavity

**Table 2:** The comparison of bIOP, CCT, and the three biomechanical parameters of NPS with IOP-matched scenario between keratoconus group (*n* = 35) and normal cornea group (*n* = 35).

Parameters	Keratoconus (*n* = 35)	Normal (*n* = 35)	*P*
bIOP [17]	13.89 ± 1.23	13.45 ± 1.02	0.142^*∗*^
Age	23.49 ± 7.05	23.66 ± 4.21	0.902^#^
CCT	489.20 ± 27.66	532.92 ± 26.64	0.000^*∗*^
AT1	6.30 ± 0.34	6.90 ± 0.32	0.000^*∗*^
DRM1	1.18 ± 0.02	1.17 ± 0.01	0.012^*∗*^
Eload	108.50 ± 9.13	90.71 ± 6.67	0.000^#^

[17] IOP from Corvis ST was corrected based on finite element modeling. Pairwise matching was performed for bIOP. ^*∗*^Two-tailed Student's *t*-test. ^*#*^Mann–Whitney *U* test.

## Data Availability

The data used to support the findings of this study are available from the corresponding author upon request.
